# The impact mechanism of geopolitical risks on ESG performance: The moderating effects of investor attention and government subsidies

**DOI:** 10.1371/journal.pone.0311659

**Published:** 2025-01-16

**Authors:** Pengfei Cheng, Mengzhen Wang, Kanyong Li, Baekryul Choi, Wenliang Chen

**Affiliations:** 1 School of Economics and Management, Hubei University of Automotive Technology, Shiyan City, Hubei Province, China; 2 School of Economics and Management, Shandong Jiaotong University, Jinan, China; 3 Department of International Trade, Jeonbuk National University, Jeonju-si, Jeollabuk-do, Republic of Korea; 4 College of Inland Open Economics, Guizhou University of Commerce, Guiyang, Guizhou, China; University of Science and Technology of China, CHINA

## Abstract

The impact of geopolitical risks (GPR) on enterprises is significant, yet the existing literature lacks a comprehensive understanding of how GPR affects environmental, social, and governance (ESG) performance. This study addresses this gap by analysing data from Chinese enterprises over the period 2009 to 2021. It empirically examines the impact of GPR on ESG performance and explores the underlying mechanisms. Specifically, the analysis considers the roles of investor attention and government subsidies as moderating factors. The results indicate that GPR inhibits corporate ESG performance. State-owned enterprises are found to mitigate these adverse effects, while privately-owned enterprises tend to exacerbate them. Mechanism tests reveal that GPR negatively impacts ESG performance by increasing financing constraints and reducing financial performance. Furthermore, increased investor attention and government subsidies can alleviate the negative effects of GPR on ESG performance. These findings offer valuable insights for organisations, governments, and stakeholders, enabling them to better respond to GPR and achieve sustainable development.

## 1 Introduction

Sustainability requires companies to focus not only on financial performance but also on the long-term development of their business while taking on social and environmental responsibilities. Environmental, social, and governance (ESG) metrics reflect an enterprise’s impact on the environment, society, and corporate governance [1, 2]. A company’s ESG strategy is considered a critical driver of sustainable development [3]. Consequently, ESG performance, as an essential criterion for measuring corporate sustainable development, has garnered increasing attention from investors, companies, and governments [4]. Companies are increasingly obtaining ESG ratings, and scholars have conducted substantial research on the impact of ESG [5]. For instance, a company’s ESG strategy can enhance stock investment returns [6], increase corporate profits [7], and reduce corporate risks [8].

Although stakeholders widely recognise the importance of ESG, the existing literature pays little attention to the factors that determine ESG performance. Only a few studies have attempted to identify the factors influencing differences in companies’ ESG performance [9]. For example, Hong et al. (2012) demonstrate that financial constraints significantly affect corporate sustainability [10], while Hamdi et al. (2022) identify corporate income as a crucial driver of ESG performance growth [11]. Unfortunately, most research ignores the impact of macro factors on ESG performance.

Notably, countries worldwide are increasingly affected by instability, with one significant factor being growing geopolitical risks (GPR) [12]. GPR is listed as one of the top five global business threats [13], manifesting as ethnic and religious conflicts, economic dependence, political instability, military intervention, armed threats, major power intervention, terrorism, and other threats to national sovereignty and military security [14]. Recent crises, such as the U.S.–China trade war and the Russia–Ukraine war, have highlighted the significant impact of geopolitical risks. Investors, the financial sector, and the media also regard GPR as critical to investment decisions [15].

In recent years, GPR has become a prominent research topic among scholars. For investors, rising GPR increases investment uncertainty, affecting profitability [16]. Consequently, increased GPR reduces local investment, inhibits trade, and decreases economic productivity [17]. However, in today’s globalised world, most international economic activities, such as trade and investment, are conducted by enterprises [18]. The impact of GPR on enterprises is thus crucial and should not be disregarded. For instance, GPR may cause economic turbulence, increasing firms’ financing costs [19]. Therefore, GPR inevitably affects the ESG performance of enterprises.

Specifically, geopolitical tensions might affect environmental performance by making resource acquisition more difficult, altering environmental policies, or neglecting environmental standards [20]. Regarding social performance, political conflicts may result in societal instability, impacting the workforce, community relations, and supply chains, thereby affecting the fulfilment of social responsibilities [21]. Concerning governance performance, political instability or policy changes may affect corporate governance structures, compliance requirements, and leadership transitions, impacting governance efficacy [22].

In summary, GPR has emerged as a critical factor influencing corporate ESG performance. Regrettably, the existing literature’s understanding of GPR’s impact on ESG performance remains limited [23]. According to stakeholder theory and institutional theory, investors and governments play a pivotal role in enterprises’ responses to risk shocks and ESG practices [24]. Therefore, it is necessary to include investor concerns and government subsidies in the analytical framework of GPR’s impact on ESG. This study aims to furnish further understanding and insights regarding this relationship.

It is particularly interesting to study the relationship between GPR and ESG performance in emerging countries. Compared to developed nations, emerging countries have lower ESG development levels, prompting governments to support companies’ ESG strategies to achieve sustainable development goals. Additionally, companies in emerging countries often have higher ownership concentration, which may impact stakeholders differently [25]. Emerging countries are also frequently at the centre of geopolitical conflicts [26]. Therefore, a more detailed study of GPR’s impact on corporate ESG performance in emerging countries is necessary.

As the largest emerging country, China is at a crucial stage of economic transformation. Given that ESG aligns with the requirements of sustainable development, China places significant importance on corporate ESG performance. Furthermore, as China’s international stature grows, its geopolitical strategies and actions, including border issues, maritime rights, and the Belt and Road Initiative, continuously evolve, offering a rich array of cases for analysing geopolitical risks [27]. As the world’s second-largest economy, China’s scale and rapid growth epitomise emerging market characteristics [28]. Studying China provides a valuable comparative perspective to understand the economic and political dynamics of other rapidly developing nations [29]. Thus, using China as the research object enhances understanding of GPR’s impact on ESG performance in emerging countries.

This study uses data from China over the period 2009 to 2021 to empirically analyse the impact of GPR on ESG. The findings indicate that GPR inhibits corporate ESG performance and adversely affects ESG in the eastern and central regions. State-owned enterprises mitigate the adverse impact of GPR, whereas privately-owned enterprises amplify these effects. Mechanism tests show that GPR hurts corporate ESG by enhancing financing constraints and reducing corporate asset returns. Additionally, increased investor attention and government subsidies can alleviate the negative impact of GPR on ESG performance.

The main contributions of this study are as follows:

1. The existing literature primarily focuses on the economic consequences of ESG performance, such as financial outcomes, investment decisions, and market value, while paying little attention to the determinants of ESG performance, especially macro factors. This study enriches the existing body of knowledge by exploring the impact of GPR on ESG performance, an area that has been relatively underexplored. This paper delves deeper into the mechanisms and heterogeneous effects of GPR on ESG performance, contributing to a more nuanced understanding of the complex relationship between geopolitical dynamics and corporate responsibility and governance. These insights are valuable for businesses, investors, and policymakers navigating the intricate landscape of ESG performance amid geopolitical uncertainties.
2. A unique aspect of this research is the analysis of the moderating roles of investor attention and government subsidies in the relationship between GPR and corporate ESG performance. The findings indicate that increased investor attention and government subsidies can mitigate the negative impact of GPR on ESG performance. This provides actionable insights for businesses, investors, and policymakers, offering strategies to bolster corporate resilience and commitment to ESG standards amid geopolitical pressures. This expands theoretical models of risk management and corporate strategy under geopolitical uncertainty, emphasising the importance of collaborative efforts by businesses, governments, and other stakeholders in achieving sustainable development goals.
3. This study focuses on China, the largest emerging economy, during a critical stage of economic transition, thereby contributing to the literature on emerging economies. By examining how GPR affects ESG performance in China, the study provides insights applicable to other emerging economies facing similar challenges. This has broader implications for understanding global corporate governance and sustainability models amidst geopolitical uncertainties. Studying China, with its rapid economic growth and significant geopolitical activities, offers a valuable comparative perspective to understand the economic and political dynamics of other rapidly developing nations.

This paper is structured as follows: In Section 2, we conduct a literature review and formulate the research hypotheses. Section 3 presents our empirical study’s methodology and variable descriptions. Section 4 presents the empirical results, while Section 5 concludes the paper.

## 2 Literature review and hypothesis development

### 2.1 GPR and ESG performance

Over the past 20 years, geopolitical risks and conflicts worldwide have continued to escalate. Countries are increasingly facing terrorism, wars, and political tensions [30]. The uncertainty caused by GPR significantly impacts the economy, politics, trade, and stock markets and also affects corporate behaviour and decision-making [14, 31]. For instance, rising GPR leads to diminished investor sentiment and a contraction in available capital for investment, thereby altering corporate behavioural decisions. This is because an increase in GPR signifies greater investment risk, prompting investors to prefer holding cash, consequently reducing the financial resources available to firms [32]. This phenomenon can be interpreted using real options theory. During periods of elevated GPR, firms prefer to adopt a ’wait-and-see’ approach due to high asymmetric adjustment costs, postponing actions until uncertainties are resolved [33]. Overall, the impact of GPR on ESG performance cannot be neglected. Drawing on existing literature and relevant theories, GPR will affect corporate ESG performance in the following areas:

Environmental performance: Geopolitical tensions often make accessing natural resources a challenge. A nation might restrict the export of scarce resources or access to foreign firms. For instance, geopolitical tensions could lead to instability in the supply of critical materials such as rare earth elements, affecting industries such as electronics and clean energy [34, 35]. Moreover, political upheaval or regime change may lead to rapid changes in environmental policies and regulations, increasing uncertainty for business operations. For example, a country might suddenly raise emission standards or revoke previous environmental protection policies. Additionally, in regions of political instability or conflict, environmental protection is often neglected, exposing firms to ethical and legal risks such as illegal logging and pollution [36, 37].

Social performance: Political conflicts or instability may trigger societal turmoil, impacting the labour market and community stability. For instance, ethnic conflicts or political protests may disrupt business operations, affecting employee safety and productivity [38]. Furthermore, GPR increases the likelihood of supply chain disruptions. Conflicts or trade restrictions might lead to shortages of raw materials, increasing costs and delaying production [39].

Governance performance: Geopolitical changes may lead to alterations in the legal and regulatory framework, increasing compliance costs and complexity. For example, a new government might implement stricter anti-corruption laws or alter corporate governance rules [40]. Additionally, political instability may result in turnover within a company’s leadership, particularly in state-owned enterprises or industries closely linked with the government [41]. Accordingly, we propose Hypothesis 1 as follows:

H1: GPR inhibits the growth of corporate ESG performance.

### 2.2 GPR, financing constraints and ESG performance

GPR has significantly altered the behaviour of economic actors and businesses, negatively impacting investments and other economic activities [42]. One critical aspect of this impact is the increased financing constraints faced by firms due to GPR. This phenomenon can be understood through several mechanisms.

Firstly, GPR leads to a contraction in credit markets. Under high GPR, banks and other financial institutions may reduce lending or increase loan costs to compensate for heightened risks [43]. Secondly, GPR induces an increase in investor risk aversion. Investors may decrease or withdraw investments due to concerns about political instability or policy changes, resulting in increased financing costs for firms [44]. Thirdly, GPR promotes market volatility and decreases capital liquidity. Geopolitical tensions may trigger market fluctuations, exacerbating information asymmetry between financial institutions and firms. This increases the cost of corporate financing and reduces capital availability [45]. Modern corporate finance theory explains that information asymmetry and agency issues make external financing more expensive than internal financing, leading to financing constraints [46]. Consequently, the rise in GPR may impose stricter financial constraints on firms.

Furthermore, financing constraints significantly affect firms’ ESG performance. Specifically, in terms of environmental performance, financing constraints might limit firms’ investments in environmental projects and sustainable technologies, which often require substantial upfront investments. With inadequate funding, firms may delay or scale down these long-term, cost-intensive environmental projects [47]. Additionally, due to the high risk and low return characteristics of ESG initiatives, traditional financial institutions are mostly reluctant to finance firms’ ESG strategies [48]. This reluctance forces firms to invest more to meet the banks’ financing scrutiny [49], but the incurred costs may not yield commensurate returns [50]. Therefore, from a cost-benefit perspective, firms also tend to reduce ESG investments [51].

In terms of social performance, activities such as community investment and employee training might be reduced due to budget constraints. Financing constraints may lead firms to cut staff or lower labour standards to reduce expenses [52, 53]. Regarding governance performance, financing constraints can exacerbate governance issues. For instance, firms may reduce compliance investments or make decisions that sacrifice long-term stability under short-term pressures [54, 55]. Based on this, we propose Hypothesis 2 as follows:

H2: GPR inhibits ESG performance growth by increasing corporate financing constraints.

### 2.3 GPR, financial performance and ESG performance

According to resource dependency theory, firms, as open systems, rely on external environmental resources. When geopolitical risks (GPR) lead to obstructed resource acquisition or increased costs, businesses may need to reallocate resources, sacrificing long-term ESG investments to maintain short-term survival and financial performance [56, 57]. Existing literature indicates that macroeconomic changes negatively impact company earnings, especially during adverse events [58]. A rise in GPR can trigger negative reactions throughout the entire economic system [59], thereby impacting firms’ financial performance [60]. Specifically, political instability or policy changes might restrict market entry through trade barriers, tariffs, or sanctions [61]. Consequently, firms may also face increased operating costs, including higher insurance premiums, compliance costs, and security expenditures [58]. Furthermore, political conflict or uncertainty can directly affect the value of firms’ assets in specific regions, leading to asset impairment [62]. Additionally, political risk might cause market demand fluctuations, thereby impacting sales and revenue [63].

According to stakeholder theory, declining financial performance can alter how a firm balances the needs of different stakeholders, particularly prioritising those groups most crucial to the company’s financial support in the short term [64]. Moreover, the Trade-Off Theory posits that, all else being equal, lower profitability levels lead to higher bankruptcy risks and increased debt costs [65]. When firms are underperforming financially, they might lack the funds and energy to engage in ESG activities, implying that a company’s financial performance is a significant determinant of ESG investment.

In terms of environmental performance, financial pressure might compel firms to cut back on environmental investments or delay implementing eco-friendly projects due to their substantial initial outlay and longer payback period. Additionally, to save costs, firms might opt for lower-cost materials or production methods with greater environmental impact [11]. Regarding social performance, under economic pressure, firms might reduce spending on social responsibility projects, such as community services, employee welfare, and training. Furthermore, to cut costs, firms might lay off workers or lower labour standards, potentially harming relations with employees and the community [66]. Declining financial performance might also lead firms to seek short-term benefits in governance, such as reducing transparency, weakening regulatory compliance, or adopting aggressive accounting strategies to "beautify" financial statements. These practices might damage the firm’s reputation and diminish trust among investors and other stakeholders [33, 67].

In conclusion, geopolitical risks significantly impact firms’ financial performance, which in turn affects their ESG investments. Political instability and policy changes can restrict market access, increase operating costs, and cause asset impairments, leading to reduced financial performance. This reduction in financial performance forces firms to prioritise short-term survival over long-term ESG investments. Consequently, firms may cut back on environmental projects, social responsibility initiatives, and governance practices. This complex interplay between GPR, financial performance, and ESG performance underscores the importance of understanding the broader macroeconomic and political context in which firms operate. Based on these considerations, we propose Hypothesis 3 as follows:

H3: GPR inhibits the growth of ESG performance by reducing corporate financial performance.

### 2.4 The moderating effects of investor attention and government subsidies

In the face of risk shocks, not only the efforts of enterprises but also those of governments and investors play an important role [68]. Therefore, in this section, we analyse the moderating effects of investor attention and government subsidies on GPR based on existing research and related theories.

With increasing societal emphasis on sustainable development and responsible investment, an ever-growing number of investors are beginning to assess corporate ESG performance as a part of their investment decisions [69]. Drawing on existing research and relevant theories, investor attention will modulate the impact of GPR on ESG in several ways. First, increased risk sensitivity arises because attention to ESG may heighten investors’ sensitivity to geopolitical risks, which often correlate with severe ESG issues such as environmental degradation, social turmoil, or governance failures [70]. Second, enhanced risk management occurs as investors focused on ESG are likely to prefer investing in firms capable of effectively managing geopolitical risks and demonstrating strong ESG performance. This encourages firms to bolster their risk management and response strategies, thereby diminishing the potential impact of GPR [71]. Third, improved transparency and communication, according to signalling theory, occurs when firms send positive signals to the market by enhancing their ESG performance, especially in high-risk environments. With increased investor attention, firms might augment their transparency and communication frequency regarding ESG, including disclosing their risk exposures and how they manage and mitigate these risks [72]. Finally, strengthened internal management and innovation are underscored by risk management theory, which highlights how firms protect corporate value by identifying, assessing, and addressing risks, including managing geopolitical risks through improved ESG performance. Firms may enhance their ESG-related internal management, including environmental management systems, social responsibility programs, and corporate governance structures [73]. Moreover, investor pressure can inspire firms to innovate, developing new technologies or business models to reduce the impact of geopolitical risks [74].

In summary, the rising attention of investors can mitigate the adverse effects of GPR on corporate ESG by enhancing firms’ risk sensitivity, improving risk management, increasing transparency and communication, and promoting internal management and innovation. Accordingly, we propose Hypothesis 4 as follows:

H4: The increase in investor attention will reduce the negative impact of GPR on ESG performance.

The institutional theory posits that corporate behaviour and decision-making are not only driven by shareholders but also influenced by the government [75]. The government is indispensable for promoting the flow of information, reducing external risk shocks, and supporting the market’s healthy development [67]. Therefore, government support is crucial in promoting ESG. Government subsidies, as a vital policy instrument, include direct financial grants, tax incentives, low-interest loans, and research and development support. These subsidies aim to reduce operational costs for businesses and support the development of specific sectors or technologies. Through subsidies, governments can encourage investment in environmental protection, social welfare, and robust governance structures, especially when faced with the uncertainties and resource constraints brought about by GPR.

Specifically, government subsidies can alleviate financial pressure by providing firms with the necessary funding to alleviate the economic pressure caused by geopolitical risks, enabling them to maintain or enhance their commitment to environmental stewardship and social responsibilities [76]. Additionally, subsidies can incentivise investment and innovation by lowering the barriers for firms investing in new technologies and projects, particularly those that enhance the firm’s ESG performance. This may include clean energy technologies, waste management systems, and social welfare programs [77]. Moreover, with financial support, firms can enhance their risk management capabilities, such as by diversifying supply chains, investing in less risky regions, or improving internal governance structures [46].

In conclusion, government subsidies, as a significant policy tool, can play a critical role when firms face GPR, helping them to alleviate the economic pressures of risks and encouraging continued investment in areas related to ESG. Accordingly, we propose Hypothesis 5 as follows:

H5: Government subsidies will reduce the negative impact of GPR on ESG performance.

**Fig 1:**
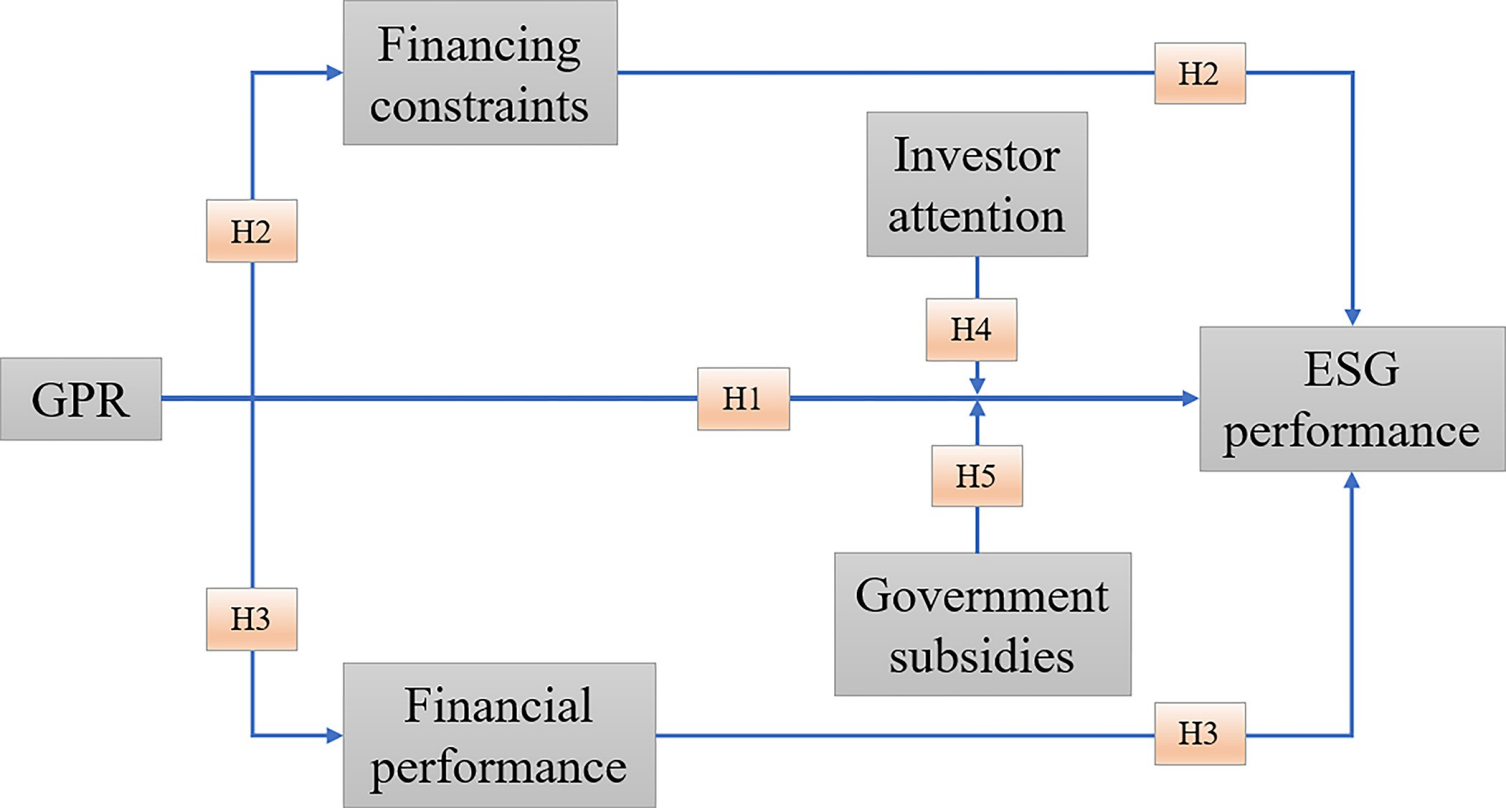
Technology roadmap.

In summary, we posit that GPR is a significant factor affecting ESG performance. Specifically, GPR can influence ESG performance by impacting the financing constraints and financial performance of enterprises. Additionally, in the process where GPR affects ESG performance, investor attention and government subsidies play crucial moderating roles. This highlights the multifaceted impact of GPR and the importance of external factors in mitigating its effects. The technology roadmap of this research is shown in Fig 1.

## 3 Methodology specification and variable description

### 3.1 Model construction

The following econometric model examines the relationship between corporate GPR and ESG performance while controlling for firm and industry fixed effects. Given that the GPR index is a time series variable, its value is the same for all companies within the same year. Therefore, controlling for the year-fixed effect would cause the GPR index to completely overlap with the year-fixed effect, making it impossible to distinguish the impact of GPR [78]. To address this issue, we do not control for the year-fixed effect. This methodological choice is consistent with previous studies by Gulen and Ion (2016) [79], Nguyen and Phan (2017) [60], and Lee and Wang (2021) [19], who also did not control for the year-fixed effect.

Furthermore, to ensure the robustness of our results, we cluster robust standard errors at the firm level. Variables are logarithmically transformed (except for ratio variables) to mitigate concerns about serial correlation and heteroskedasticity. This comprehensive approach ensures that our model accurately captures the relationship between GPR and ESG performance, providing reliable and valid results.

$${ESG}_{it}=\partial_{0}+\partial_{1}{GPR}_{t}+\partial_{2}{\sum Controls}_{it}+\mu_{i}+\gamma_{j}+\varepsilon_{it}$$ (1)

where i represents the enterprise and t represents the year. ESG_it_ represents the ESG performance index; GPR_t_ represents the geopolitical risk index; Controls_it_ represents the set of control variables; μ_i_ is the firm effect; γ_j_ is the industry fixed effect; and ε_it_ represents the error term.

Mediation models allow us to intricately explore the complex relationships between variables and provide a more detailed understanding of the underlying mechanisms. In testing mediation effects, much academic research has traditionally drawn on the causal step regression method proposed by Baron and Kenny (1986) [80]. This method offers an intuitive and clear analytical logic for mediation effects, facilitating explanation by researchers and comprehension by readers. However, in recent years, the validity and procedural appropriateness of this method have been questioned by many scholars [81]. In response to these concerns, Zhao et al. (2010) [82] have extensively discussed the procedures for testing mediation effects and recommended the Bootstrap method proposed by Preacher and Hayes (2004) [83] as a more reliable alternative. Consequently, our research, grounded in theoretical analysis, hypothesises that geopolitical risks impact corporate ESG performance through specific mediating pathways, namely financing constraints and corporate financial performance.

To test our hypotheses, we employed the causal step regression method for mediation effect analysis as proposed by Baron and Kenny (1986) [80]. Additionally, we conducted tests using the Bootstrap method to ensure robustness and accuracy in our findings. The mediation effect model is outlined as follows. This comprehensive approach enables us to validate our theoretical framework and provides robust insights into the mediating effects of financing constraints and corporate financial performance on the relationship between geopolitical risks and ESG performance.

$${ESG}_{it}=\alpha_{0}+{\alpha_{1}GPR}_{t}+{\alpha_{2}\sum Controls}_{it}+\mu_{i}+\gamma_{j}+\varepsilon_{it}$$ (2)

$$M_{it}=b_{0}+{b_{1}GPR}_{t}+{b_{2}\sum Controls}_{it}+\mu_{i}+\gamma_{j}+\varepsilon_{it}$$ (3)

$${ESG}_{it}=c_{0}+{c_{1}GPR}_{t}+{c_{2}M}_{it}+{c_{3}\sum Controls}_{it}+\mu_{i}+\gamma_{j}+\varepsilon_{it}$$ (4)

where M_it_ includes financing constraint (KZ_it_) and financial performance (ROV_it_).

Based on theoretical analysis and research hypotheses, we added the interaction term of GPR to the baseline model to test the moderating effects of investor attention and government subsidies on GPR.

$${ESG}_{it}=\partial_{0}+{\partial_{1}GPR}_{t}+{\partial_{2}Z}_{it}+{\partial_{3}{GPR}_{t}\times Z}_{it}+{\partial_{4}\sum Controls}_{it}+\mu_{i}+\gamma_{j}+\varepsilon_{it}$$ (5)

where Z_it_ includes investor attention (IA_it_) and government subsidies (GOV_it_).

### 3.2 Variable description and data source

The sample of this study consists of China’s A-share listed companies from 2009 to 2021. After excluding companies with incomplete sample data, we obtained 29,976 observations. The basic information and financial data of the companies in this study are sourced from the CSMAR database.

1. Explained variable: To evaluate companies’ ESG performance, we use Huazheng ESG Ratings, known for their coverage and data completeness, and widely used by scholars [84]. These ratings can be accessed at Huazheng ESG Ratings (https://www.chindices.com/).
2. Core explanatory variable: The GPR index used in this study is measured based on of Caldara and Iacoviello’s (2022) GPR index [85]. Caldara and Iacoviello (2022) [85] constructed this index by calculating the number of articles related to GPD (e.g. military threats, terrorism, internal insurgency and external aggression) as a proportion of the total number of articles in newspapers [86]. It is a composite index that includes all internal and external shocks related to war and military tensions [87]. Since news stories are a timely and reliable source of information for investors, this index has been widely used in recent studies [88, 89]. The index comes from the EPU database (https://www.policyuncertainty.com/gpr.html).
3. Mediating variable: Mediating variables include financing constraints and firm financial performance. We choose the KZ index as a proxy for financing constraints. The larger the KZ index, the greater the financing constraints faced by the company. We choose return on assets (ROA) as a proxy for a company’s financial performance [90].
4. Moderating variable: Moderating variables include investor attention and government subsidies, which is the sum of various subsidies the government provides to enterprises. Internet search volume can directly reflect how much a stock attracts investors’ attention [91]. Most relevant research on China’s investor concerns uses the Baidu Index. Because Baidu is China’s mainstream online search engine [92], Chinese investors mostly use it to search for relevant information. Therefore, we use the Baidu Index compiled with stock names as keywords as a proxy for investor attention [93]. The data was extracted from the CSMAR database (https://www.gtarsc.com/).
5. Control variables: Referring to existing research, our control variables include ① company size (SIZE): Measured as the natural logarithm of total assets. ② shareholding ratio of the largest shareholder (SR): Measured as the percentage of shares held by the largest shareholder. ③ total operating income (TOI): Measured as the natural logarithm of total operating income. ④ tangible asset ratio (TAR): Measured as the ratio of tangible assets to total assets. ⑤ leverage (LEV): Measured as the ratio of total liabilities to total assets [94]. All control variable data come from the CSMAR database.

Table 1 presents the basic statistical analysis of each variable. The standard error of ESG performance is 5.720, indicating significant differences in corporate ESG performance. Additionally, the highest and lowest values of GPR are 0.917 and 0.379, respectively, showing substantial fluctuations in GPR during the sample period, which provides a basis for our analysis.

**Table 1:** Variables described.

Variables	Abbreviation	Unit	Mean	Std. Dev.	Min	Max
Environment, society, and governance	ESG	Index	72.65	5.720	36.62	92.93
GPR index	GPR	Index	0.572	0.211	0.379	0.917
Financing constraints (KZ index)	KZ	Index	1.030	2.440	-11.33	13.66
Return on Assets	ROA	%	0.040	0.670	-30.69	108.4
Government subsidies	GOV	100 million yuan	1.200	5.680	0	301.8
Investor attention index	IA	%	12.66	0.730	6.230	17.29
Company size	SIZE	100 million yuan	22.16	1.340	13.76	28.64
Shareholding ratio of the largest shareholder	SR	%	34.03	14.97	0.290	89.99
Total operating income	TOI	%	21.50	1.520	9.040	28.72
Tangible asset ratio	TAR	%	0.920	0.090	0.110	1
Leverage	LEV	%	0.450	0.590	-0.190	58.08

## 4 Findings and discussions

### 4.1 Baseline results

Table 2 presents the regression results of the baseline model. In Columns 1–6 of Table 2, the coefficients for GPR are all significantly negative. Specifically, in Column 6, the coefficient of GPR is -0.007. This indicates that for every 1% increase in GPR, a company’s ESG performance decreases by 0.007%. These findings confirm our expectations that GPR suppresses the growth of ESG performance, thus confirming Hypothesis 1.

**Table 2:** Baseline results.

	(1)	(2)	(3)	(4)	(5)	(6)
	ESG	ESG	ESG	ESG	ESG	ESG
GPR	-0.006***	-0.016***	-0.012***	-0.007***	-0.007***	-0.007***
	(0.002)	(0.002)	(0.002)	(0.002)	(0.002)	(0.002)
SIZE		0.010***	0.010***	0.003*	0.004**	0.004**
		(0.001)	(0.001)	(0.002)	(0.002)	(0.002)
SR			0.026***	0.025***	0.024***	0.021***
			(0.003)	(0.003)	(0.003)	(0.003)
TOI				0.007***	0.007***	0.009***
				(0.002)	(0.002)	(0.002)
TAR					0.013**	0.012*
					(0.007)	(0.007)
LEV						-0.020***
						(0.002)
Constant	4.279***	4.056***	3.961***	3.972***	3.965***	3.911***
	(0.001)	(0.023)	(0.026)	(0.029)	(0.028)	(0.030)
Firm fixed	YES	YES	YES	YES	YES	YES
Industry fixed	YES	YES	YES	YES	YES	YES
*R* ^2^	0.493	0.498	0.503	0.506	0.506	0.513
N	29976	29976	29976	29976	29976	29976

Note: Standard errors in parentheses

*, ** and *** represent significance levels at 10%, 5% and 1%, respectively.

This result aligns with existing research. For instance, Klymenko et al. (2021) suggest that increased uncertainty and risk may force companies to prioritise financial performance over ESG investing [95]. Similarly, Bauer et al. (2022) argue that ESG strategies require substantial investments of corporate funds and resources [96], which may not yield immediate economic benefits, making firms more cautious and likely to reduce ESG investments during periods of high uncertainty.

There are several possible explanations for this outcome. First, most companies are primarily profit-driven. When confronted with the high uncertainty brought about by rising GPR, companies must focus on survival and satisfying shareholder expectations, often prioritising financial performance over ESG investments [97]. Second, ESG strategies require significant investment of corporate funds and resources but may not provide immediate economic benefits. This puts companies at a disadvantage in market competition [98]. Furthermore, ESG initiatives may result in a winner-take-all scenario, where companies with an initial advantage dominate the market [99]. Faced with greater risks, companies may adopt more cautious strategies and reduce investment. Therefore, in times of high uncertainty brought by GPR, companies may reduce their ESG activities.

### 4.2 Heterogeneity analysis

Table 3 presents the results of the heterogeneity test. First, we divided the sample into eastern, central, and western regions to examine regional heterogeneity in the impact of GPR on ESG performance. The results, shown in Columns 1–3 of Table 3, indicate that enterprises in the eastern and central regions are significantly adversely affected by GPR. This may be due to the fact that, compared to the western regions of China, the eastern and central areas are more developed, with a higher concentration of industries, financial markets, and international trade activities. Due to these regions’ greater exposure to international markets and complex supply chains, they might be more sensitive to geopolitical tensions. Although the western region is still developing, its economic structure is distinct. Given its industrial focus and lower degree of international participation, it may not be directly affected by GPR [100].

**Table 3:** Heterogeneity analysis.

	(1)	(2)	(3)	(4)	(5)	(6)
	ESG	ESG	ESG	ESG	ESG	ESG
	(Eastern)	(Central)	(Western)	(X = SOE)	(X = POE)	(X = FOE)
GPR	-0.007**	-0.011**	-0.005	-0.018***	0.004	-0.007***
	(0.002)	(0.005)	(0.005)	(0.003)	(0.003)	(0.002)
SIZE	0.007**	0.005	-0.004	0.004*	0.004**	0.004**
	(0.002)	(0.005)	(0.004)	(0.002)	(0.002)	(0.002)
SR	0.027***	0.014**	0.009	0.019***	0.019***	0.021***
	(0.004)	(0.007)	(0.008)	(0.003)	(0.003)	(0.003)
TOI	0.008***	0.009**	0.013***	0.009***	0.009***	0.009***
	(0.002)	(0.004)	(0.004)	(0.002)	(0.002)	(0.002)
TAR	0.010	0.028**	-0.001	0.009	0.010	0.012*
	(0.008)	(0.011)	(0.021)	(0.007)	(0.007)	(0.007)
LEV	-0.023***	-0.019***	-0.012**	-0.019***	-0.019***	-0.020***
	(0.002)	(0.004)	(0.004)	(0.002)	(0.002)	(0.002)
X				0.020***	-0.007	0.005
				(0.005)	(0.004)	(0.011)
ESG* X				0.026***	-0.023***	-0.010
				(0.003)	(0.003)	(0.0111)
Constant	3.849***	3.901***	4.056***	3.913***	3.921***	3.912***
	(0.037)	(0.067)	(0.059)	(0.029)	(0.030)	(0.030)
Firm fixed	YES	YES	YES	YES	YES	YES
Industry fixed	YES	YES	YES	YES	YES	YES
*R* ^2^	0.514	0.495	0.499	0.516	0.516	0.514
N	20491	5094	4391	29976	29976	29976

Note: Standard errors in parentheses

*, ** and *** represent levels of significance at 10%, 5% and 1%, respectively.

Second, we examined the heterogeneous effects of firm ownership, focusing on state-owned enterprises, private-owned enterprises, and foreign-owned enterprises. Our findings reveal that state-owned enterprises mitigate the adverse impact of GPR, while private-owned enterprises exacerbate the negative effects of GPR. This outcome may be attributed to several factors. First, state-owned enterprises often establish good relationships with stakeholders from their inception, making it easier to obtain financial and technical support. This is not typically the case for non-state-owned enterprises, which tend to face higher financing constraints. Since banks are still the main credit providers in China’s financial market, loan availability from state-owned banks is uneven [9]. Additionally, due to the weak foundation of most non-state-owned enterprises [101], these enterprises may experience more significant negative impacts when facing GPR shocks. Second, in the Chinese context, state-owned enterprises bear more social responsibilities due to their political attributes, while the primary goal of private-owned enterprises is to maximise profits [102]. This finding aligns with Hsu et al. (2021) [103], who demonstrated that state-owned enterprises play more significant environmental and social roles than other enterprises. Therefore, when GPR rises, state-owned enterprises are more likely to implement ESG behaviours to assume environmental and social responsibility.

### 4.3 Mechanism analysis

Table 4 reports the results of the mediation effect regression. Columns 1–2 of Table 4 show that GPR increases the KZ index and decreases ROA. The results in Columns 3–4 demonstrate that the KZ index inhibits ESG performance, while ROA promotes it. Furthermore, the Bootstrap test results indicate that the mediating effect of financing constraints is significant, with the interval (LLCI = 0.0013, ULCI = 0.0020) not including 0, and the size of the mediation effect (a×b) being 0.0016. The mediating effect of financial performance is also significant, with the interval (LLCI = -0.0007, ULCI = -0.0002) not containing 0, and the size of the mediation effect (a×b) being -0.0003. This validates the results of our causal step regression mediation model. In other words, GPR can inhibit the growth of ESG performance by increasing corporate financing constraints and reducing corporate financial performance, thereby verifying Hypotheses 2 and 3. Alsagr and van Hemmen (2021) [104] and Bilgin et al. (2020) [86] have explored the broader economic and investment impacts of geopolitical risks, finding that heightened GPR can lead to increased market volatility and reduced investment. Our research extends these findings by showing that GPR not only affects market behaviour but also directly influences corporate ESG performance by increasing financing constraints and reducing financial performance.

**Table 4:** Mechanism analysis results.

	(1)	(2)	(3)	(4)
	KZ	ROA	ESG	ESG
GPR	0.562***	-0.041**	-0.004**	-0.007***
	(0.047)	(0.019)	(0.002)	(0.002)
SIZE	-0.504***	-0.016	0.003*	0.004**
	(0.052)	(0.015)	(0.002)	(0.002)
SR	-0.455***	-0.004	0.019***	0.021***
	(0.067)	(0.025)	(0.003)	(0.003)
TOI	-0.283***	0.039***	0.008***	0.009***
	(0.047)	(0.010)	(0.002)	(0.002)
TAR	0.136	-0.053	0.012*	0.012*
	(0.168)	(0.036)	(0.006)	(0.007)
LEV	2.548***	-0.108**	-0.018***	-0.020***
	(0.055)	(0.039)	(0.002)	(0.002)
KZ			-0.001**	
			(0.000)	
ROA				0.003*
				(0.001)
Constant	22.73***	-0.559	3.949***	3.912***
	(0.904)	(0.350)	(0.032)	(0.030)
Firm fixed	YES	YES	YES	YES
Industry fixed	YES	YES	YES	YES
*R* ^2^	0.680	0.103	0.569	0.568
N	29976	29976	29976	29976
Bootstrap test				
	Indirect effects	LLCI	ULCI	P-value
GPR→KZ→ESG	0.0016	0.0013	0.0020	0.000
GPR→ROA→ESG	-0.0003	-0.0007	-0.0002	0.008

Note: Standard errors in parentheses

*, ** and *** represent significance levels at 10%, 5% and 1%, respectively.

Specifically, against the backdrop of frequent geopolitical events, the economic environment and financial markets are filled with uncertainty. Investors may postpone or reduce their investments, resulting in reduced capital liquidity, which increases the financing costs for enterprises [105]. Additionally, as GPR rises, investors may demand higher risk premiums, further increasing companies’ external financing costs [106]. On the other hand, the financial performance of enterprises is also impacted by increased uncertainty [107]. Corporate ESG investments increase a company’s total costs without achieving clear financial benefits. Therefore, when confronted with increased financing constraints and reduced financial performance brought about by GPR, companies tend to reduce their ESG activities [108]. Li et al. (2023) also obtained similar results [109].

### 4.4 Moderating effect analysis

Table 5 reports the results of the moderation effect analysis. The coefficients of the interaction terms between government subsidies and investor attention are positive and significant, indicating their moderating role. The influence of external stakeholders, particularly investors, on corporate behaviour has been extensively studied. For instance, Amel-Zadeh and Serafeim (2018) found that investors increasingly use ESG information to make informed investment decisions [70]. Boffo and Patalano (2020) highlighted the critical role of government policies in promoting ESG investments [110]. Our study corroborates these findings, showing that increased government subsidies and investor attention can reduce the adverse impact of GPR on ESG performance, thereby verifying Hypotheses 4 and 5.

**Table 5:** Moderating effect analysis results.

	(1)	(2)
	ESG	ESG
GPR * GOV	0.008***	
	(0.001)	
GPR * IA		0.015***
		(0.002)
GPR	-0.145***	-0.203***
	(0.017)	(0.029)
GOV	0.005***	
	(0.001)	
IA		-0.001
		(0.002)
SIZE	0.003*	0.007***
	(0.002)	(0.002)
SR	0.024***	0.021***
	(0.003)	(0.003)
TOI	0.010***	0.010***
	(0.002)	(0.002)
TAR	0.007	0.005
	(0.007)	(0.007)
LEV	-0.023***	-0.022***
	(0.002)	(0.002)
Constant	3.796***	3.824***
	(0.033)	(0.042)
Firm fixed	YES	YES
Industry fixed	YES	YES
*R* ^2^	0.513	0.534
N	29976	29976

Note: Standard errors in parentheses

*, ** and *** represent significance levels at 10%, 5% and 1%, respectively.

This outcome can be explained by several factors. Firstly, investors’ time and energy are limited, and their ability to process information is constrained [111]. When more investors pay attention to a particular stock, the demand for the stock increases in the short term, driving up the stock price [112]. Consequently, greater investor attention can reduce information asymmetry between companies and investors, mitigating the financing constraints imposed by GPR and improving corporate capital gains [113, 114].

Secondly, firm policies and actions communicated directly to investors and stakeholders are crucial for enhancing ESG performance. Government subsidies can enhance the legitimacy of enterprises, inject additional impetus into their development, boost the confidence of enterprises and investors, mitigate the adverse effects of GPR, and provide robust support for the continued implementation of ESG strategies [115].

### 4.5 Robustness test

We verify the robustness of our regression results using the following methods:

1. Lag period analysis: Referring to Gozgor et al.’s (2022) method [43], we use one lag period of the GPR index for robustness testing. The results are shown in Column 1 of Table 6.
2. Variable replacement: First, we use the GPR historical (GPRH) data provided by the EPU database. Caldara and Iacoviello (2021) constructed this index using three newspapers starting in 1990 [85]. Thus, we replace the raw GPR data with GPRH for robustness testing. Second, we use the political stability and absence of violence/terrorism index from the World Bank WDI database as a proxy for GPR. The results are shown in Columns 2 and 3 of Table 6.
3. Sample period adjustment: To remove the impact of the COVID-19 pandemic, we reduce the sample period to 2009–2019. The results are shown in Column 4 of Table 6.
4. Addition of macro control variables: We incorporate macro control variables as suggested by Feng et al. (2023) [116]. The results are shown in Column 5 of Table 6.
5. Endogeneity adjustment: We use the two-stage least squares (2SLS) method to address potential endogeneity problems. Selecting appropriate instrumental variables involves ensuring they are related to the endogenous explanatory variable and affect the dependent variable only through this variable [117]. We choose China’s military expenditure (% of GDP) and the political stability and absence of violence/terrorism index as instrumental variables. China’s military expenditure might reflect the country’s response to the external security environment and geopolitical strategy, influencing regional or global GPR, but it does not directly correlate with firms’ ESG performance [118]. The results are shown in Column 6 of Table 6.

**Table 6:** Robustness test results.

	(1)	(2)	(3)	(4)	(5)	(6)
	ESG	ESG	ESG	ESG	ESG	ESG
GPR	-0.005**	-0.009***	-0.009**	-0.005**	-0.003*	-0.005***
	(0.002)	(0.002)	(0.004)	(0.002)	(0.002)	(0.001)
SIZE	0.005**	0.004**	0.003*	0.004**	0.007***	0.004***
	(0.002)	(0.002)	(0.002)	(0.002)	(0.002)	(0.001)
SR	0.021***	0.022***	0.023***	0.013***	0.020***	0.022***
	(0.003)	(0.003)	(0.003)	(0.003)	(0.003)	(0.002)
TOI	0.010***	0.006***	0.008***	0.007***	0.008***	0.009***
	(0.002)	(0.002)	(0.002)	(0.002)	(0.002)	(0.001)
TAR	0.008	0.011*	0.011	0.026***	0.014**	0.012**
	(0.007)	(0.007)	(0.007)	(0.007)	(0.007)	(0.004)
LEV	-0.021***	-0.020***	-0.020***	-0.016***	-0.021***	-0.020***
	(0.002)	(0.002)	(0.002)	(0.002)	(0.002)	(0.001)
GDP					0.092***	
					(0.017)	
OPEN					-0.007***	
					(0.002)	
Under-identification test						22000
						[0.000]
Weak identification test						61000
Sargan statistic						0.302
						[0.583]
Constant	3.873***	3.917***	3.934***	3.997***	3.887***	3.923***
	(0.032)	(0.029)	(0.028)	(0.031)	(0.043)	(0.017)
Firm fixed	YES	YES	YES	YES	YES	YES
Industry fixed	YES	YES	YES	YES	YES	NO
N	26433	29976	29976	23493	29976	29976

Note: Standard errors in parentheses; P-values in square brackets

*, ** and *** represent significance levels at 10%, 5% and 1%, respectively.

The results in Columns 1–5 of Table 6 demonstrate that in various robustness tests, GPR is significantly negatively correlated with ESG, consistent with the baseline model results. Furthermore, in Column 6, the under-identification test, weak instrumental variable test, and Sargan statistical results indicate that our 2SLS regression results are valid. The GPR coefficient remains significantly negative, suggesting that our baseline model does not suffer from serious endogeneity problems, and the results are robust.

## 5 Conclusions

This study significantly advances the understanding of the impact of geopolitical risks (GPR) on corporate ESG performance, focusing on Chinese A-share listed companies from 2009 to 2021. The results confirm that an increase in GPR significantly inhibits corporate ESG performance. This finding underscores the detrimental effects of geopolitical uncertainties on sustainable corporate practices, providing businesses with critical insights to better anticipate and mitigate these adverse impacts. Specifically, the study delineates the mechanisms through which GPR affects ESG performance, revealing that GPR increases corporate financing constraints and reduces financial performance. These insights emphasise the need for companies and policymakers to address financial barriers and maintain robust financial health to support ESG activities. Moreover, the study demonstrates that enhanced investor attention and government subsidies can mitigate the negative effects of GPR on ESG performance. This finding provides actionable strategies for businesses and policymakers to bolster corporate resilience. Encouraging greater investor engagement in ESG issues and providing targeted government support can help firms sustain their ESG commitments even amid geopolitical uncertainties. Theoretically, this research enriches existing models by integrating GPR and external moderating variables into frameworks for corporate sustainability and risk management. Practically, the insights gained are valuable for business leaders, investors, and policymakers aiming to navigate the complexities of ESG performance amid geopolitical uncertainties.

Based on the research results, we recommend that, first, whether relating to enterprises, governments or other stakeholders, attention should be paid to the adverse impact of rising GPR on corporate ESG behaviours. Both enterprises and governments should formulate relevant development strategies to hedge against the negative impact of GPR. Second, as far as enterprises are concerned, they should focus on improving their reputational value, proactively disclosing their information to the public, and improving their reputation and image to obtain more financial and policy support. Third, the government should fully recognise the role of government subsidies and provide financial support to forward-looking companies to support corporate ESG behaviours evidenced by practical actions. For example, through various incentive measures, companies are encouraged to improve their ESG performance under the impact of GPR.

While this study enriches the research on the impact of GPR on ESG performance, it has some limitations. First, our research sample is limited to Chinese A-share listed companies from 2009 to 2021, excluding small and medium-sized enterprises. Second, given the exposure of companies to international markets and global supply chains, a firm’s international positioning (such as being a multinational corporation or exporter) may significantly influence how they perceive and manage GPR. Further analysis to ascertain whether a company is a multinational or exporter might prove valuable; however, due to data constraints, such an analysis was not feasible. Finally, if developed countries were used as the research object, the results might differ from those of emerging countries. Thus, comparative analysis could lead to interesting findings.

## Supporting information

S1 Data (XLS)

## List of abbreviations

ESG: Environmental, social, and governance
GPR: Geopolitical risk
IA: Investor attention
GOV: Government subsidies
KZ Index: Kaplan-Zingales index (Financing constraints
ROA: Return on assets
SIZE: Company size
SR: Shareholding ratio of the largest shareholder
TOI: Total operating income
TAR: Tangible asset ratio
LEV: everage
